# Structural pharmacology of Chinese medicine: technological breakthroughs in decoding multi-target synergy and precision mechanisms

**DOI:** 10.1007/s44307-026-00095-x

**Published:** 2026-03-13

**Authors:** Caiyan Wang, Yanling Li, Lin Zhuo, Songzhu Xu, Mengyao Wang, Zhongqiu Liu

**Affiliations:** 1 State Key Laboratory of Traditional Chinese Medicine Syndrome, International Institute for Translational Chinese Medicine, Guangzhou University of Chinese Medicine, Guangzhou, 510006 China; 2 Chinese Medicine Guangdong Laboratory (Hengqin Laboratory), Depth Cooperation Zone in Hengqin, Guangdong-Macao In, Zhuhai, 519000 China

**Keywords:** Structural Pharmacology of Chinese Medicine (SPCM), Multi-target Synergy, TCM Modernization

## Abstract

Traditional Chinese Medicine (TCM) offers valuable therapeutic strategies for chronic and infectious diseases, yet the inherent complexity of its multi-component, multi-target formulations and synergistic effects presents substantial challenges to pharmacological mechanistic understanding. Structural pharmacology of Chinese Medicine has emerged as a transformative discipline, integrating structural biology, computational chemistry, and pharmacology to elucidate the precise mechanisms underlying TCM efficacy. This review synthesizes technological advancements that enable the characterization of synergistic mechanisms and dynamic molecular interactions in TCM. Key advancements include high-resolution structural techniques such as X-ray crystallography, cryo-electron microscopy, sophisticated computational approaches such as AI-driven predictive modeling, and advanced analytical platforms. We critically examine persistent technical hurdles, such as capturing transient binding events and modeling complex multi-component system dynamics. Finally, we outline future research trajectories to establish a predictive and adaptable scientific foundation for TCM modernization, facilitating its evidence-based global integration and application in precision medicine.

## Introduction

### Historical significance and modern challenges of TCM

Traditional Chinese medicine (TCM) is increasingly integrated into global healthcare systems. It plays a pivotal role in managing chronic and infectious diseases, contributing to a more robust global healthcare network. The discovery and application of *Artemisinin* exemplify TCM's contributions. *Artemisinin*, extracted by You-you Tu's team, has been called the "greatest breakthrough in tropical medicine in the twentieth century." Due to its unique mechanism of action and absence of cross-resistance with traditional antimalarial drugs, it has become a core antimalarial agent globally. WHO reports indicate that *Artemisinin*-based combination therapies benefit over 200 million malaria patients annually, substantially reducing malaria deaths from 736,000 in 2000 to 409,000 in 2019, and saving millions of lives worldwide. In 1920, ephedrine was isolated from *Ephedra sinica*, marking the first alkaloid of Chinese medicinal origin and a milestone in the pharmacology of natural medicine. As a prototype β-adrenergic agonist, it served as the basis for bronchodilators that effectively relieve acute asthma and chronic obstructive pulmonary disease, and it remains relevant in respiratory emergency medicine. Berberine, derived from Coptis chinensis and Phellodendron amurense, is among the first natural orally hypoglycemic compounds. It improves insulin resistance by activating the AMPK pathway and is cited in the Chinese Guidelines for the Prevention and Control of Type 2 Diabetes Mellitus as an alternative to metformin. It is also used as a dietary supplement for the treatment of metabolic syndrome in the U.S. and Europe.These examples highlight the potential of TCM-derived products in treating major diseases and represent remarkable cases of TCM inheritance and innovation.

However, the very success of isolating single, potent natural products like artemisinin also inadvertently underscored a fundamental challenge in TCM research. TCM formulations are inherently complex, typically comprising multiple chemical components that work synergistically. Their therapeutic effects arise from the synergistic interactions of these components, rather than from individual constituents alone. The 'single component-single target' paradigm, which is effective for studying isolated natural products, struggles to capture the holistic and network-based pharmacology of full TCM preparations. Therefore, while the reductionist approach of isolating active ingredients is valuable, it simultaneously reveals the limitation of such methods in elucidating the complex, multi-component, multi-target synergistic system that defines TCM therapy. This realization creates a pressing need to leverage modern scientific and technological approaches.

It is crucial to distinguish between isolated natural products and traditional Chinese medicine preparations. Isolated natural products, such as artemisinin, ephedrine, and berberine, are single chemical entities derived from medicinal sources. Their study often follows the 'single component-single target' paradigm, which has been fruitful for drug discovery. In contrast, TCM preparations, typically used as complex multi-herb formulations, exert their therapeutic effects through the synergistic interactions of numerous chemical components. This multi-component system targets a network of biological pathways, representing a holistic approach that is fundamentally different from the reductionist model often applied to single natural products. The primary challenge and focus of Structural Pharmacology of Chinese Medicine (SPCM) lie in deciphering the complex synergy within these multi-component preparations, rather than merely analyzing individual constituents in isolation.

TCM formulations are inherently complex, typically comprising multiple chemical components. Their therapeutic effects arise from the synergistic interactions of these components, rather than individual constituents alone. Furthermore, TCM components undergo dynamic metabolism in the body, generating a wide range of structurally complex metabolites. Traditional research methods struggle to accurately track and characterize these metabolites. Significantly, the efficacy mechanism involves a complex "multi-component-multi-target" synergistic regulatory network, in contrast to the simple "single component-single target" model. This has led to greater difficulties in analyzing the structures and dynamic metabolites of complex components in drug studies. Therefore, there is a pressing need to leverage modern scientific and technological approaches, particularly advanced technologies such as digital technologies, artificial intelligence (AI), and big data analytics, to gain insight into the composition, pharmacological effects, internal mechanisms, and clinical applications of TCM. To date, more innovative technologies are essential to advancing the modernization of TCM research and development, ultimately providing novel therapeutic strategies to address complex and refractory diseases.

### Structural pharmacology as a bridge

**Fig. 1:**
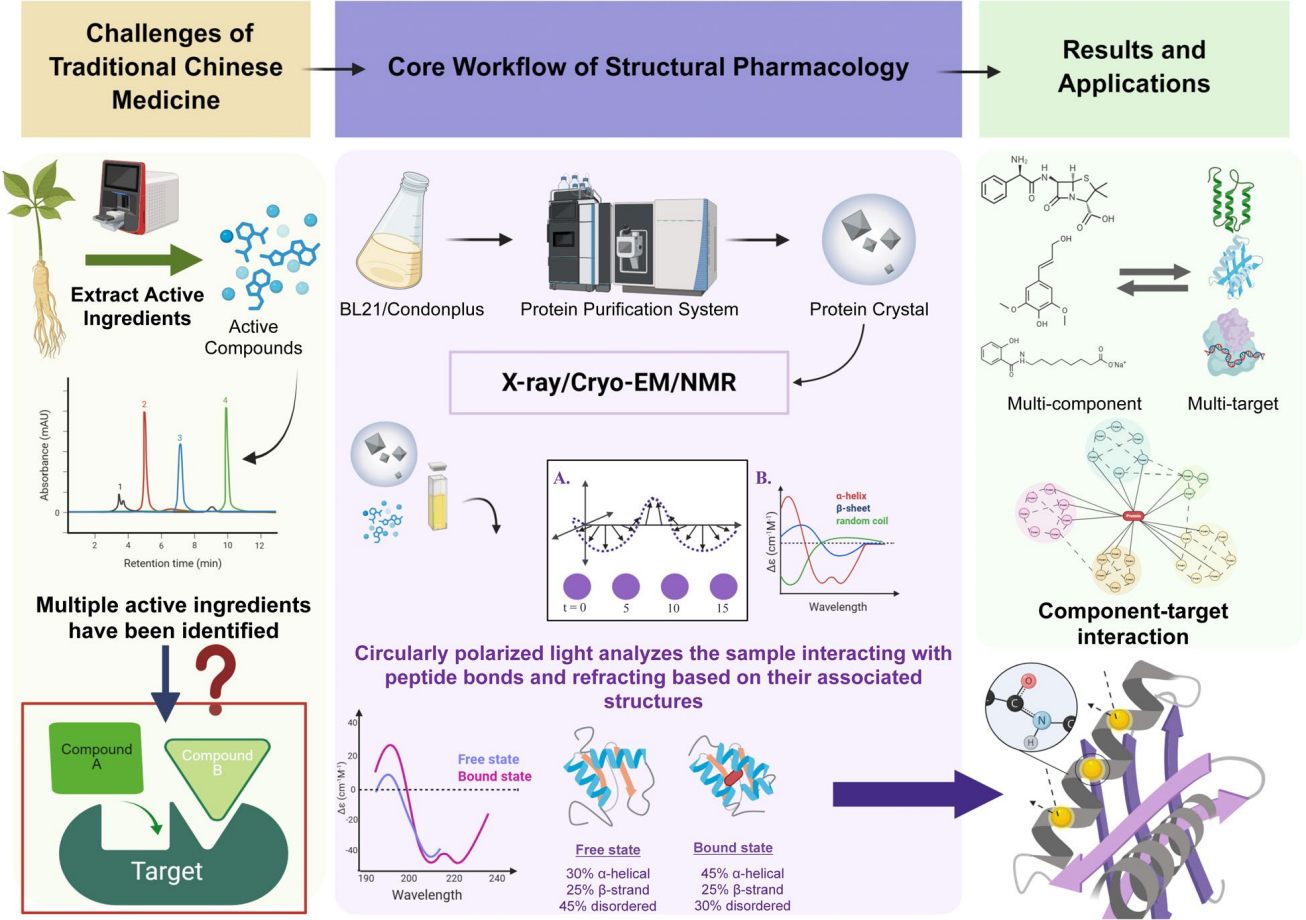
Structural pharmacology as a bridge. This figure vividly illustrates the bridging function of structural pharmacology in connecting TCM with modern drug research. On the left side, it presents the characteristics of TCM, including multi-component chemical substances and multi-target action points, as well as demand to clarify the scientific basis of "multi-target synergy". On the right side, it shows the technical workflow of structural pharmacology, while explicitly listing key experimental equipment such as cell crushers, protein purification systems, and single crystal X-ray diffractometers. It highlights that structural pharmacology can improve the efficiency of active ingredient discovery

Structural pharmacology is an interdisciplinary field that employs structural biology approaches to advance pharmacological research, elucidating drug action mechanisms at an atomic resolution and establishing a foundation for rational drug design. Primary methodological frameworks include macromolecular crystallography, computational virtual screening, and structure-based drug design, with principal research foci encompassing the structural characterization of disease-relevant therapeutic targets and de novo drug development. Its core objective is to systematically analyze the interaction mechanisms between drugs (or ligands) and biomacromolecules (such as receptors, enzymes, and ion channels) using high-resolution structural analysis techniques (e.g., cryo-electron microscopy, X-ray crystallography) and computational simulation methods (e.g., molecular docking, free energy calculations). This approach directly enhances pharmacological understanding and guides drug optimization design. As illustrated in Fig. 1 (Structural Pharmacology as a Bridge), this interdisciplinary paradigm specifically serves as a bridge connecting Traditional Chinese Medicine (TCM)-characterized by multi-component chemical substances and multi-target interaction points-with modern drug research. By demonstrating its technical workflow, it aims to improve the discovery efficiency of active components in traditional Chinese medicine.

#### High resolution structural technology

High-resolution structural techniques are primarily employed in the early stages of drug discovery, where the resolution of drug-target complex structures helps address the critical question of "how drugs bind to their targets." The high-resolution crystal structure of monkeypox virus I7L protease provides both structural insights and a lead compound for developing broad-spectrum antiviral drugs targeting orthopoxviruses. In Traditional Chinese Medicine (TCM) research, structural analysis of epimedium polysaccharides lays the foundation for drug development (Li et al., 2015). X-ray crystallography determines protein three-dimensional structures by analyzing diffraction patterns from X-ray penetration through protein crystals, significantly advancing drug development based on protein-small molecule binding patterns. However, this technique requires proteins to form crystals, which limits its practical applications. Cryo-electron microscopy (Cryo-EM) enables imaging and 3D structural analysis of biomacromolecules in near-physiological conditions. Since the 'resolution revolution,' Cryo-EM has resolved over 30% of G protein-coupled receptor (GPCR) structures compared to X-ray crystallography alone, providing critical insights into GPCR-mediated signaling pathways and drug development. However, Cryo-EM equipment is expensive and data processing is complex. Nuclear magnetic resonance (NMR) offers atomic-level resolution in solution environments, but its application is limited by the resolution of large molecular weight complexes.

#### Computational and network methods

Computational and network-based approaches are primarily employed during the drug design phase to address the challenge of "how to design more effective drug molecules" through computer simulations, thereby reducing experimental costs and time. Computational methods such as molecular docking, molecular dynamics simulations, and quantum chemical calculations can predict drug-target binding patterns, affinity, and dynamic interactions, revealing thermodynamic and kinetic mechanisms to support virtual screening and drug design. For instance, during the COVID-19 pandemic, molecular docking technology was rapidly applied to screen potential drug molecules targeting SARS-CoV-2 proteins. However, due to the complexity of biological systems, simulation results may deviate from actual conditions. Network pharmacology integrates high-throughput data integration, database searches, data mining, and target prediction technologies to systematically demonstrate complex relationships between TCM components and their targets. Through systems like the Traditional Chinese Medicine (TCM) database system (e.g., Batman-TCM), information on TCM active ingredients and their potential targets can be obtained. The component-target interaction network serves as a key tool for analyzing the synergistic effects of TCM's multi-component system.

#### Methodological comparison and integration: revealing the synergistic mechanism of Traditional Chinese Medicine (TCM)

Network pharmacology and structural pharmacology complement each other methodologically (Fig. 1). Network pharmacology excels at providing holistic, top-down perspectives by constructing system-level networks that connect TCM components, targets, and diseases, effectively generating hypotheses about TCM mechanisms of action. However, its predictions are typically correlational and lack atomic-level structural details. In contrast, structural pharmacology offers a bottom-up mechanistic research approach. By analyzing the three-dimensional structures of drug-target complexes and simulating their dynamic interactions, it provides visual and quantitative evidence for network pharmacology predictions, establishing causal "structure–activity relationships" and "structure–function relationships" at the molecular level. In short, network pharmacology reveals which components may interact with which targets, while structural pharmacology elucidates how these interactions precisely occur in three-dimensional space and why they lead to functional changes. Therefore, these two fields are highly complementary: network pharmacology provides a "roadmap" of potential interactions, while structural pharmacology offers high-resolution mechanistic validation for key nodes on this roadmap.

#### Integrated application: decoding multi-target synergy mechanism

The therapeutic efficacy of Traditional Chinese Medicine (TCM) often stems from the synergistic regulation of multiple components targeting diverse biological targets. Structural pharmacology integrates these mechanisms through three key approaches: 1) Target-Component Interaction Network: By combining structural data of individual components and targets, we construct "component-target-pathway" networks to analyze correlations between core components and key targets. 2) Allosteric Regulation Mechanism: TCM components may modulate downstream signaling by binding to non-active sites (allosteric sites) of targets, thereby altering their conformational states. Structural pharmacology techniques enable visualization of conformational changes before and after ligand binding, combined with molecular dynamics simulations to trace allosteric effect pathways (e.g., residue-distance variations and hydrogen bond reorganization). In studies exploring how the natural product ergosterol improves fatty liver and insulin resistance, researchers discovered that Atractylodin II (AT II), an active component in Chinese herbal medicine, specifically activates DGKQ through gender-specific conformational changes (Zheng et al. 2024), confirming DGKQ as the target protein of AT II.To systematically address the complexity of the "multi-component, multi-target" system, structural pharmacology has developed an integrated research pathway that progresses layer by layer and forms a closed-loop validation process. Firstly, network pharmacology is employed to screen high-value core interaction pairs from the vast array of component-target relationships. Subsequently, structural biology technique-such as X-ray crystallography, Cryo-EM, and NMR-are used to resolve the complex structures formed by key components and their targets, thereby elucidating the dominant modes of action. Further, molecular dynamics simulations are applied to investigate the dynamic interactions between multiple components and single or multiple targets, revealing their synergistic mechanisms—such as conformational selection and induced-fit—on a temporal scale. Finally, the acquired structural and dynamic information is integrated with pharmacological efficacy data from cellular and animal models, establishing a complete causal chain from atomic structure to overall pharmacological activity. This approach enables a multi-dimensional and integrated interpretation of the synergistic mechanisms underlying Traditional Chinese Medicine.The study on the traditional Chinese medicine formulation "Huashi Baidu Formula" exemplifies a systematic approach to elucidating its anti-SARS-CoV-2 mechanisms (Xu et al. 2023). Initial characterization of the formula's chemical composition was conducted using ultra-performance liquid chromatography coupled with quadrupole time-of-flight mass spectrometry (UPLC-Q-TOF/MS), identifying 343 distinct chemical constituents. Subsequent analysis revealed 60 prototype compounds and 66 metabolites as systemically absorbed components, establishing the potential pharmacologically active basis. Among these, six bioactive constituents—magnolol, emodin, and quercetin, among others—demonstrated synergistic antiviral effects through distinct target engagement. Mechanistic investigations determined that quercetin and licochalcone A primarily inhibit SARS-CoV-2 main protease (Mpro), whereas neoglycyrol and glycyrol A target RNA-dependent RNA polymerase (RdRp). Furthermore, structural analysis elucidated that neoglycyrol and glycyrol A exert inhibitory effects on phosphodiesterase 4 (PDE4) through occupation of its catalytic active site.

In summary, structural pharmacology provides visual and quantitative evidence for the 'multi-component, multi-target' mechanism of action in Traditional Chinese Medicine (TCM). By analyzing the interactions between TCM components and their targets, this approach enables the construction of multi-component synergistic networks, representing a significant shift in the field from empirical description to precise molecular mechanism analysis.

### Scope and innovation of this review

**Fig. 2:**
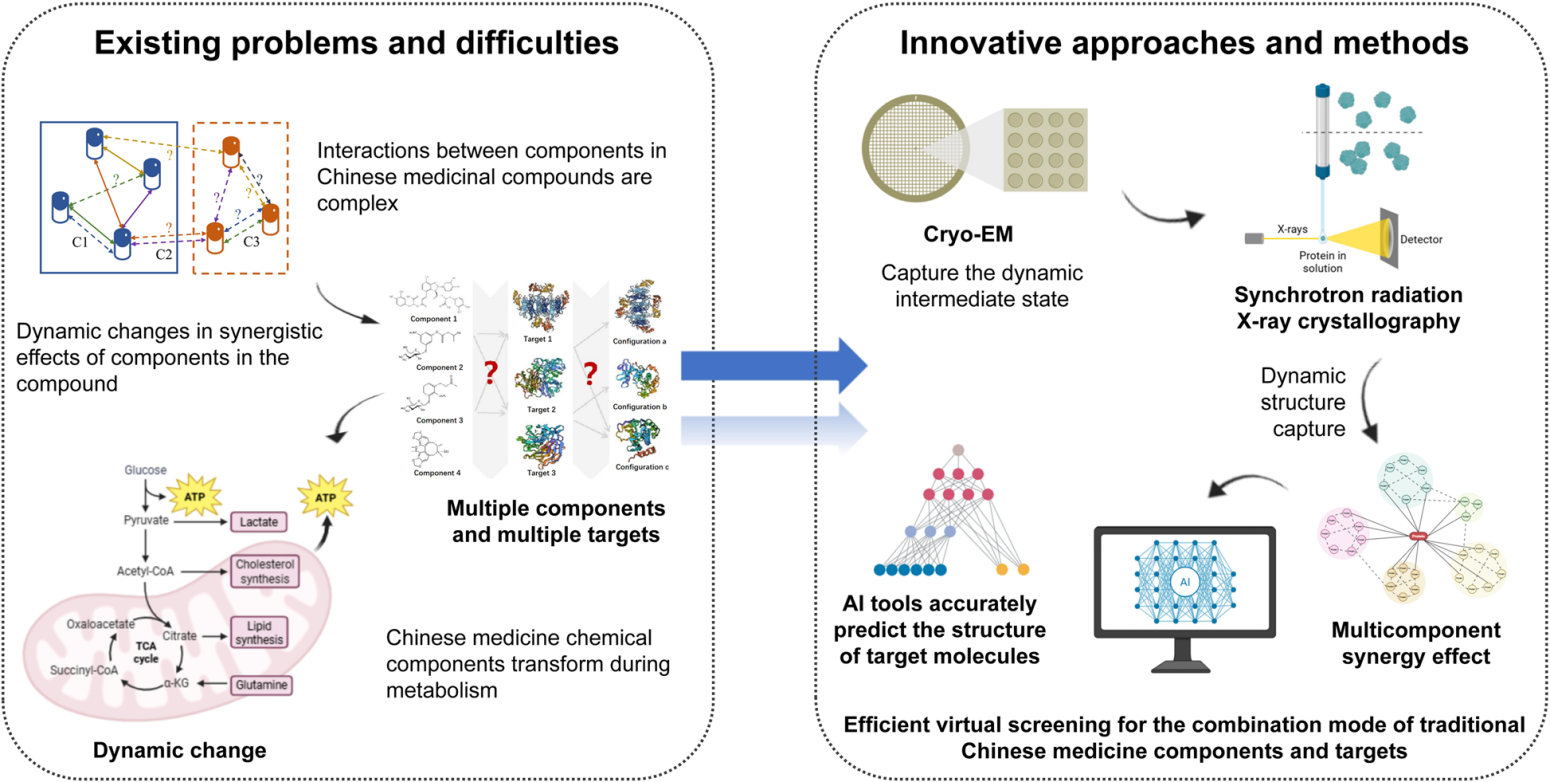
Scope and innovation of this review. This figure focuses on the core challenges and innovative solutions in the research of Chinese medicine compound formulations and intuitively demonstrates the role of structural pharmacology in breaking through traditional research paradigms. The left side clearly lists three key challenges (as visible in the figure). On the right side, targeted innovative technical solutions are presented. These solutions provide technical support for deciphering the multi-component-multi-target synergistic mechanisms underlying TCM formulations

This review aims to provide a comprehensive synthesis of how SPCM is transforming TCM research (Fig. 2). We transcend traditional descriptive approaches by focusing on three innovative dimensions that form the core of this review: (i) the critical role of structural dynamics in understanding in vivo molecular interactions, (ii) a systems-level analysis of multi-component synergistic effects based on molecular interaction networks, and (iii) the transformative potential of AI-driven reverse analysis for target discovery and compound screening. By synthesizing advances across these fronts, we outline a future trajectory for establishing a predictive and adjustable scientific foundation for TCM modernization, thereby facilitating its evidence-based global integration.

## Bioactive molecules and synergistic architectures of TCM

### Major classes of bioactive molecules

The complex pharmacological effects of Chinese medicines arise from an elaborate network of active molecules that achieve multidimensional recognition and regulation of biological targets through their unique chemical architecture and spatial conformation. According to molecular weight, the active molecules of TCM are mainly categorized into two core systems: small-molecule compounds and macromolecular compounds.Beyond characterizing known active constituents, structural pharmacology provides a rational framework for discovering novel active pharmaceutical ingredients from complex TCM formulations. By analyzing three-dimensional structural features essential for successful target engagement—such as spatial arrangements of specific functional groups, key rigid planar conformations, and electronic distribution properties—researchers can identify privileged structural scaffolds. This enables virtual screening of TCM compound libraries for molecules exhibiting structural similarity or containing these critical scaffolds, even when derived from distinct herbal sources. Furthermore, elucidating the binding modes of multiple components to a single target (e.g., an enzyme's active site and its allosteric site) reveals how structurally diverse molecules synergistically regulate the same target or pathway through cooperative interactions, thereby guiding the discovery of constituents with complementary bioactivity.

Based on this classification, this chapter integrates research from our group with advances by a major team in China (Jigang Wang and Kewu Zeng) to systematically elucidate the structure–function association mechanism of TCM active molecules.

#### Small-molecule compounds in Chinese medicine

**Fig. 3:**
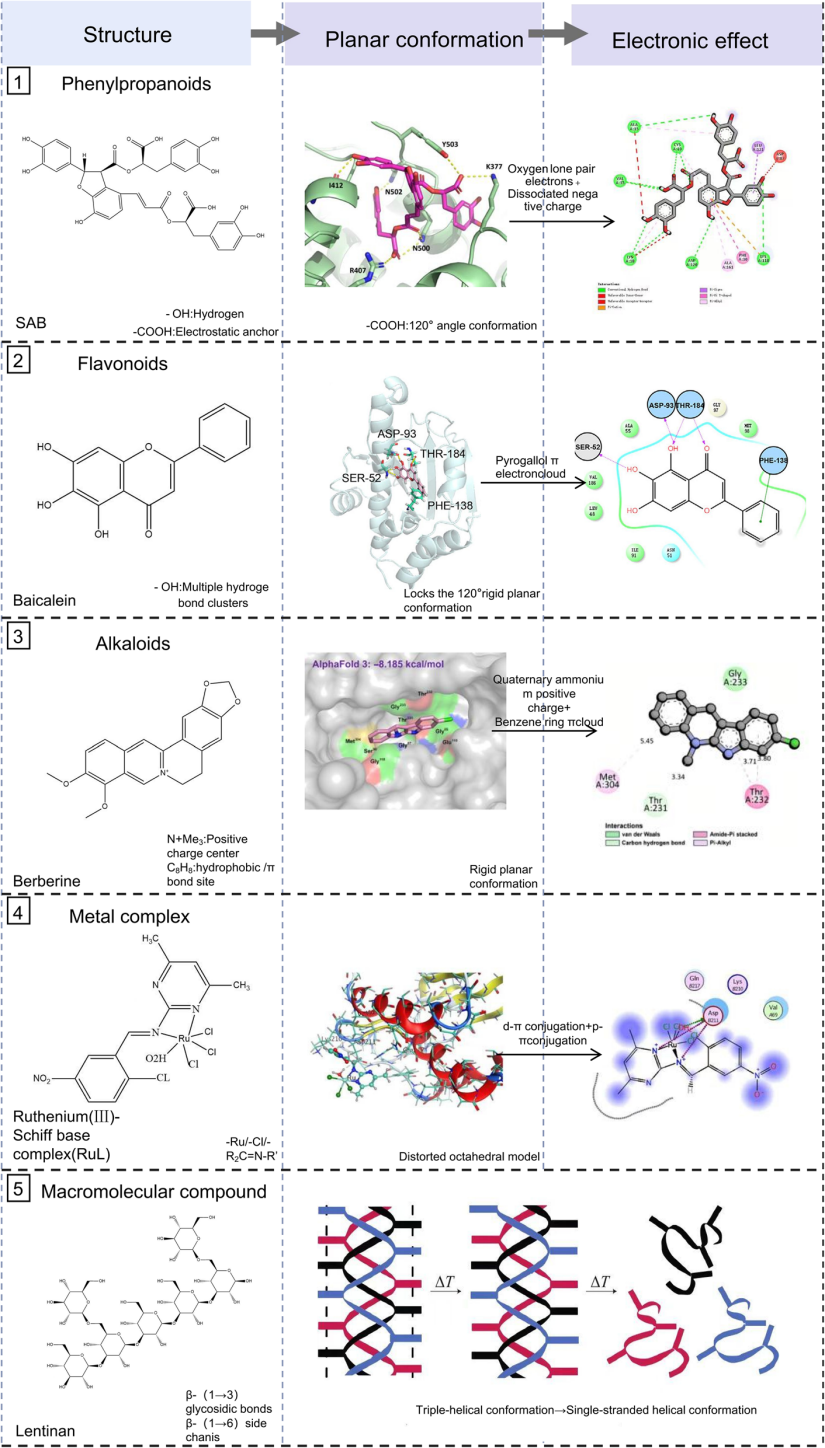
Structural characteristics, planar conformations and electronic effects of the main bioactive molecules in TCM. This figure presents the core structural traits, typical planar conformations and key electronic effects of major bioactive molecules in TCM, anchored in the "structure–function" relationship. It classifies these molecules into two classes: small-molecules and macromolecular compounds. For small molecules, it details structural skeletons, specific groups, planar arrangements and electronic effects. For macromolecules like mushroom polysaccharides, it shows advanced conformations, such as β-(1 → 3) glucan backbone with β-(1 → 6) branches forming a triple helix. It demonstrates how these molecular architectures enable precise targeting and regulation, establishing a foundation for understanding TCM active component mechanisms

Small-molecule compounds in TCM are typically defined as organic or metal–organic complexes with a molecular weight < 900 Da. Their structural diversity, small-molecule compounds in TCM, achieve specific binding to targets through the precise arrangement of functional groups, specific planar conformations, and electronic effects. They are important components of TCM active ingredients, enabling fine targeting. Structurally, diverse small molecules in TCM refer to mononuclear or polynuclear complexes with a ligand molecular weight of < 900 Da and an overall molecular weight of ≤ 1,500 Da. These compounds are typically water-soluble and capable of penetrating cell membranes (Fig. 3). Major categories include phenylpropanoids, flavonoids, alkaloids, and low-molecular-weight metal complexes. The latter exhibits dual efficacy, combining the properties of small molecules and inorganic ions through synergistic coordination between organic ligands and metal ions. In this section, we will use representative compounds as examples to systematically illustrate the intrinsic correlation between their structural features and the mechanism of target action.

##### Phenylpropanoid (PPA), an analog of benzene ring

Phenylpropanoid compounds are characterized by a C6-C3 basic skeleton, and several subtypes, such as coumarins and lignans, have been derived from it through esterification, polymerization, and other modifications. Among the many phenylpropanoid polyphenols, Salvianolic acid B (SAB) has become a paradigmatic molecule for investigating the mechanism of action of this class of compounds due to its high antioxidant potency, abundance, and stable and identifiable structure (Sun et al. 2024). SAB consists of multiple phenylpropanol units linked by ester bonds and is rich in catechol, phenolic hydroxyl, and carboxylic acid groups. Critically, its phenolic hydroxyl groups are arranged in a 120° plane angle functional group arrangement with the carboxylic acid groups, which is highly compatible with the rigid planar conformation of the transmembrane structural domain of the linker protein GJB2. In molecular recognition, the lone pair of electrons from the phenolic hydroxyl oxygen of SAB forms hydrogen bonds with amino acid residues in the transmembrane region of GJB2. Concurrently, the negative charge resulting from the dissociation of the carboxylic acid moiety creates an electrostatic attraction with the ammonium group of residue R272 in the cytoplasmic domain of GJB2.

In hepatocellular carcinoma models, abnormally localized GJB2 recruits ASB2, leading to ubiquitination and degradation of IκBα, activating the NF-κB pathway, which upregulates HIF-1α expression to enhance glycolysis and provide energy for tumor cells. Simultaneously, NF-κB activation increases PD-L1 expression, facilitating tumor immune evasion. As a small-molecule inhibitor of GJB2, SAB binds specifically to GJB2 and effectively inhibits its activity, strongly confirming the biological principle of "structure determines function" (Liu et al. 2024).

Jigang Wang's team found that Eupalinolide B (EB), a sesquiterpene lactone featuring conjugated α,β-unsaturated carbonyl groups and hydrophobic terpene rings, covalently binds to the Cys335 sulfhydryl group in branched-chain amino acid aminotransferase 1 (BCAT1) via Michael addition reaction. Meanwhile, the methyl side chain of its terpene ring inserts hydrophobically into the hydrophobic site of the BCAT1 active pocket, while the rigid planar structure formed by a five-membered lactone ring and a six-membered terpene ring complements the β-folded lamellar structure of the BCAT1 active pocket. In addition, the π-electron cloud of the α,β-unsaturated carbonyl group and the sulfur atom of the Cys335 sulfhydryl group form a conjugated system that reduces the activation energy of the addition reaction, and the electron delocalization effect within the terpene ring enhances the hydrophobicity of the molecule and stabilizes the complex through van der Waals interactions. This synergy of complementary spatial arrangement of functional groups, rigid planar conformation, and multiple electronic effects enables EB to precisely inhibit BCAT1 enzyme activity, thereby regulating BCAA metabolism and downstream oncogenic pathways (Kuang et al. 2025).

The mangostin (α-mangostin, α-MG) studied by Kewu Zeng's team is a xanthone that promotes the K48-linked ubiquitination degradation of RTN4 through a triple synergistic mechanism.

"Complementary spatial arrangement of functional groups-Rigid planar conformation-multi-electron effect". The hydroxyl group of o-triphenyltriol is distributed in a 120° plane, forming multiple hydrogen bonds with His478 and Asp512 in the E3 ubiquitin ligase UBR5. The rigid plane of the xanthone skeleton is complementary to the α-helix hydrophobicity and van der Waals complementarity of RTN4, which locks the bonding site. Additionally, the phenol oxygen atom generates additional π-electrons with the His478 imidazole group through n → π* charge transfer, enhancing the stability of hydrogen bonds This synergistic interaction triggers RTN4 ubiquitination degradation, inducing a curvature changes in the endoplasmic reticulum membrane and generating focal death "bubbles". In a mouse model of osteosarcoma, α-MG combined with anti-PD-1 significantly reduced tumor volume (Zhao et al. 2025). Using chemical proteomics, X-ray crystallography, and Cryo-EM, Jigang Wang and Kewu Zeng's team elucidated the atomic-level spatial matching mechanism between natural products and their amino acids.

##### Flavonoid

Flavonoids share a common C6-C3-C6 carbon framework, and the number and position of their phenolic hydroxyl groups are determining factors for the key structures of bioactivity (Fig. 3). Taking baicalein as an example, its o-phenyltriol hydroxyl groups are arranged in a 120° plane, enabling the formation of multiple hydrogen bonds with residues such as Ser52 and Asp93 in the ATPase domain of Hsp90. The rigid planar conformation of baicalein complements the α-helix structure of Hsp90, which allows it to be accurately embedded in the ATP-binding pocket of Hsp90. In addition, the π-π synergy between the n → π* charge transfer of the hydroxyphenolic oxygen of o-triphenyltriol and the π-π synergy of the His93 imidazole ring significantly strengthens the stability of hydrogen bonding. This synergy of hydrogen-bonding network, spatial conformational matching, and π-electronic effect competitively inhibits Hsp90 ATPase activity. The key mechanism is the inhibition of Hsp90 chaperone function, which destabilizes its client protein COX-2 and targets it for ubiquitin–proteasome degradation, leading to down-regulation of the inflammatory cascade. In a rat fever model, baicalein exerts a long-lasting antipyretic effect through this pathway (Zhang et al. 2022). Notably, the spatial orientation of the phenolic hydroxyl group of baicalein is essential for its successful insertion into the Hsp90 ATP-binding pocket and for its anti-inflammatory function; disruption of its structural integrity results in a significant decrease in activity. This mechanism reveals the tertiary structure-target-function relationship of flavonoids.

##### Alkaloid

The biological activity of alkaloids is closely related to their structural specificity. Berberine is a typical isoquinoline alkaloid that exerts its effects through a triple synergistic mechanism involving "charge-conformation-electronic effects," concurrently targeting both the AMPK-mTOR and NF-κB signaling axes. The rigid planar conformation of the conjugated benzene ring of berberine was embedded in the hydrophobic pocket of the DNA-binding domain of the p65 subunit of NF-κB, blocking its nuclear translocation. The π-electron cloud of the benzene ring and the hydrophobic site of NF-κB enhanced binding stability through van der Waals interactions. Additionally, berberine simultaneously inhibits the two pro-tumor signaling axes, AMPK-mTOR and NF-κB, in a triple synergistic "charge-conformation-electron" mode. Specifically, the positive charge of the quaternary ammonium group (-N⁺Me₃) was electrostatically complementary to that of the Glu96/Asp157 negative pocket on the surface of the AMPK subunit, which induced the formation of a hydrophobic pocket in the DNA-binding domain of the AMPK subunit. The positive charge of the conjugated benzene ring forms electrostatic complementarity with the negative charge of Glu96/Asp157 on the surface of the AMPK catalytic subunit, which induces the phosphorylation of Thr172 and the inhibition of mTOR. Simultaneously, the rigid plane of the conjugated benzene ring is embedded in the hydrophobic slit of the p65 DNA-binding domain of NF-κB, and the aryl-ring π-electron cloud generates n → π* charge transfer with residues such as Leu534, superimposed on van der Waals' forces, and blocks p65 nuclear translocation. This synergistic mechanism of "charge orientation-conformational locking-electron reinforcement" enables berberine to inhibit mTOR by activating AMPK and inhibit NF-κB independently of AMPK, thus exerting dual antitumor effects through these complementary pathways (Li et al., 2015).

##### Metal compounds

Metal coordination compounds are low-molecular-weight complexes centered on metal ions, with ligands bound by coordination bonds. Their biological activity is governed by spatial configuration, bonding mode, and charge distribution. Representative examples include tanshinone-copper (II), planar tetrahedral complexes, artemisinin-iron (II) endoperoxide-bridged octahedral complexes, and ruthenium (III)-Schiff base complexes such as RuL.

Take the ruthenium (III)-Schiff base complex (RuL) as an example: the functional groups are arranged octahedrally with the central ruthenium atom as the core, three chlorine atoms, and one water molecule in cis-coordination. The Schiff base ligand forms a four-membered chelating ring through the nitrogen atom, with nitro and chlorophenyl groups extending outward. The central ruthenium(III) has a slightly distorted octahedral configuration, with three cis-coordinated chlorine atoms and one water molecule leaving an opening in the equatorial plane. After coordinating with ruthenium, the ligand forms a near-planar structure of the pyridine ring and the benzene ring after coordination with ruthenium. Despite the slight distortion of the overall octahedral configuration, it can be adapted to the DNA double helix and the caspase-3 activation pocket, so that the near-planar unit of the pyridine-benzene ring is embedded exactly into the DNA groove or in the caspase-3 activation pocket. The near-planar unit of the pyridine-benzene ring is embedded in the DNA groove or the caspase-3 active pocket. In terms of intermolecular interactions, the ligand water molecule forms a 2.8 Å hydrogen bond with the DA6 region of DNA, while the axial chlorine atom makes van der Waals contact with nucleobases. The Schiff base nitrogen forms a 3.1 Å sulfur-nitrogen interaction with the Cys285 sulfhydryl group of caspase-3. Electronically, the d-orbital of ruthenium (III) forms a d-π conjugation with the ligand π-system, and the lone pair of electrons of the nitrogen atom forms a p-π conjugation with the sulfhydryl group. The positive charge of Ru^3^⁺ is electrostatically complementary to the positively charged regions of the DNA phospho-skeleton or of the Arg207/Lys212 region of caspase-3, and this distribution of charge makes it compatible with the positive residues of the DNA phospho-skeleton and caspase-3. This charge distribution induces electrostatic attraction to the DNA phosphate backbone and to the positively charged residues of caspase-3, thereby initiating DNA breakage and caspase-3-mediated apoptosis. Together, these two processes constitute the structural basis for its anti-tumor activity (Noureldeen et al. 2022).

These examples demonstrate that the efficacy of TCM small molecules results from precise structural features, including specific functional group placement, rigid planar conformations, and electronic effects. Such features enable targeted interactions—including hydrogen bonding, hydrophobic effects, π-π stacking, electrostatic forces, and coordination—that are essential for molecular recognition and modulation. These well-defined structure-based interactions are central to understanding the efficacy and mechanism of action of TCM and underpin rational drug design and development.

#### Macromolecular compounds in Chinese medicine

Macromolecular compounds such as polysaccharides, peptides, proteins, nucleic acids, etc., exert their biological functions through complex and sophisticated advanced three-dimensional conformations (Fig. 3). Their functional regulation is achieved through specific conformational features such as helical structure, sugar chain branching, or peptide chain folding, which can activate or regulate specific cell signaling pathways and life activity processes.

Mushroom polysaccharides, as large neutral molecules with a β-(1 → 3) main chain and β-(1 → 6) branches, adopt a stable triple-helical conformation in water. This specific structure enables complementarity with Dectin-1 on dendritic cells, initiating a signaling cascade that enhances immune function. Loss of the triple-helical form weakens this effect, directly illustrating that the defined structure underlies immune enhancement.

The contrast between conformational integrity leading to functional activation and conformational disruption resulting in inactivation directly illustrates the core logic that structure determines function. This finding shows that the higher-order spatial conformation of polysaccharides supports receptor recognition and signal transduction, which is fundamental for investigating the structure–activity relationship (SAR) of TCM polysaccharides. This serves as a paradigm for understanding the direct correlation between conformation and function in TCM polysaccharides.

To sum up, small- and medium-molecule compounds of TCM active ingredients precisely intervene on disease-related targets with structural diversity and fine molecular recognition features, while large-molecule compounds build complex immune-regulating networks with stable, three-dimensional conformations. However, they interpret the scientific essence of "structure determines function and function expresses structure" in TCM across multiple dimensions.

### Synergistic architectures in herbal formulations

#### Spatial complementarity of components in compound formulas

As the core of TCM clinical medication, the unique advantage of TCM compounding stems from the complex synergistic network formed by the multiple components of the compound through spatial complementarity and structural similarity. Multi-component complex prescriptions can generate a "1 + 1 > 2" synergistic effect through a "multi-component-multi-target-multi-pathway" regulatory mechanism. This effect can even be upgraded to the network level and more systematically demonstrate the characteristics of overall TCM regulation.Structural pharmacology provides a unique methodological framework that moves beyond phenomenological observations of synergy to deliver mechanistic insights. The integrated approach involves: First, employing network pharmacology to predict potential multi-target interactions, followed by molecular docking against three-dimensional structures of relevant targets (e.g., Mpro and RdRp) to prioritize potential synergistic pairs. Subsequently, techniques such as X-ray crystallography and cryo-electron microscopy enable direct visualization of how distinct active ingredients bind to different sites on the same target (e.g., active versus allosteric sites) or to various proteins within the same pathway, thereby providing structural evidence for complementary mechanisms of action. Finally, molecular dynamics simulations play a critical role in capturing the temporal dimension of synergy. This technique reveals how the binding of one component induces conformational changes in the target protein that enhance the binding of a second component (through "conformational selection") or stabilize specific functional states, thereby quantifying allosteric communication between binding sites. This multi-technique integration enables researchers to construct dynamic, atomic-level models elucidating how multiple components achieve a cooperative effect exceeding the sum of their individual contributions.

The classical antitumor herb pair Astragali Radix-Curcumae Rhizoma in a 2:1 ratio exhibits a paradigm of complementary spatial synergy. The identification of the core bioactive components within this herb pair, such as the flavonoid quercetin from Astragali Radix and the diphenylheptane curcumin from Curcumae Rhizoma, typically relies on integrated analytical approaches. Techniques like liquid chromatography coupled with high-resolution mass spectrometry (LC–MS) are employed for the comprehensive profiling and identification of constituents in the extract. Subsequently, affinity ultrafiltration mass spectrometry or molecular docking-based virtual screening from a constructed compound library are often used to pinpoint components with high binding affinity to specific targets of interest, such as HIF-1α in this case. Having identified these key components through such methods, their synergistic mechanism can be elucidated.The flavonoid monomer quercetin and diphenylheptane curcumin in the formula can directly bind to HIF-1α, form hydrogen bonds with α,β-unsaturated carbonyls by catechol hydroxyl and covalent interactions, respectively, and inhibit the nuclear translocation of HIF-1α. This downregulates the expression of the key glycolysis enzyme PFKFB3, reduces VE-cadherin endocytosis, and enhances endothelial cell connectivity. Meanwhile, Astragaloside IV and bisdemethoxycurcumin promoted vascular coverage by vascular smooth muscle cells and pericyte NG2 via hydrophobic interactions, increased the expression of basement membrane type IV collagen by 47%, and repaired vascular structural integrity (Liang et al. 2025). This multi-component synergistic mode from different targets demonstrates the characteristics of TCM compounding in treating tumors through multiple targets and provides a novel perspective for cancer treatment. In the ulcerative colitis model, the same compound was found to bind besides Glu443, Glu372, and Arg405 in the MD domain of Hsp90, inducing conformational changes in the adjacent NTD domain. This conformational shift further inhibits the nuclear translocation of HSF1 and downregulates the MAPK pathway (Zhao et al. 2024). In addition, the relationship with CHPF2 was identified, further validating the multi-target therapeutic potential of natural products and underscoring the broader relevance of the "structure-target-function" network in TCM.

#### Structural-based scientific explanation of the " Jun-Chen-Zuo-Shi "

As the core theory of Chinese herbal formula compounding, the scientific connotation of "Jun-Chen-Zuo-Shi" has been gradually deciphered through modern structural chemistry and molecular biology research in recent years. A groundbreaking study has revealed the intrinsic connection between this theory and the structure–function relationship of its ingredients, using Compound Huangdai Tablet as a model, and has provided a concrete molecular mechanism for interpreting "Jun-Chen-Zuo-Shi".

In the treatment of acute promyelocytic leukaemia (APL), the active ingredient in compound Huangdai Tablets contains tetrasodium arsenic tetrasulfide (As4S4). Its unique tetrahedral cage structure blocks the cancer signaling pathway by forming covalent bonds between sulphur atoms and the cysteine residues of the APL-causing protein PML-RARα (Cys369, Cys488), leading to its ubiquitination and degradation. X-ray crystallography studies show that the As4S4 cage structure can adapt to the dimerization interface of PML-RARα, forming a "molecular clamp" effect and laying the foundation for the Jun Drug's targeting effect. This establishes the drug's dominant position in targeting the core disease-causing agent. Indirubin in Indigo Naturalis forms a sulfur-bonded stabilizing complex conformation with the sulfur atom of As4S4 through the indole ring, and its planar structure is embedded in the ATP-binding pocket of CDK6, which forms a "dual-pathway regulation" with the protein degradation of the drug, increasing the efficiency of cell cycle arrest in G1 phase by 3.7 times, and assisting the drug in strengthening its antitumor effect. Tanshinone IIA of Salvia miltiorrhiza, by virtue of its phenanthrenequinone structure embedded in the hydrophobic cluster of VEGFR2 tyrosine kinase domain. It activates the Nrf2 pathway while inhibiting tumor angiogenesis and reduces intracellular reactive oxygen species levels by 63% through modulation of glutathione metabolism, effectively antagonizing the toxicity of the monarchical drug. The polysaccharide of Pseudostellaria heterophylla binds to myeloid integrin αvβ3 receptor through β-(1 → 4)-glucan to increase the accumulation of the compound in the bone marrow by 2.8 times, enabling precise delivery.

This study demonstrates that Compound Huangdai Tablet forms a multi-level therapeutic network by exploiting complementary spatial structures, electronic effects, and dynamic regulation among its ingredients. This network functionally embodies the 'Jun-Chen-Zuo-Shi' principle at the molecular level. The sovereign drug anchors the pathological target with a rigid structure; the minister drug enhances efficacy via electronic effects; the assistant drug mitigates toxicity; and the envoy drug ensures precise delivery. Together, these roles generate a synergistic effect. Importantly, this work sets a research paradigm that links molecular structure to compounding mechanism, facilitating the transition from 'principal drug and adjuvant' to a quantifiable, predictable scientific system and advancing innovative development in TCM compounding.

## Cutting-edge technologies in structural elucidation

### High-resolution structural biology tools

**Fig. 4:**
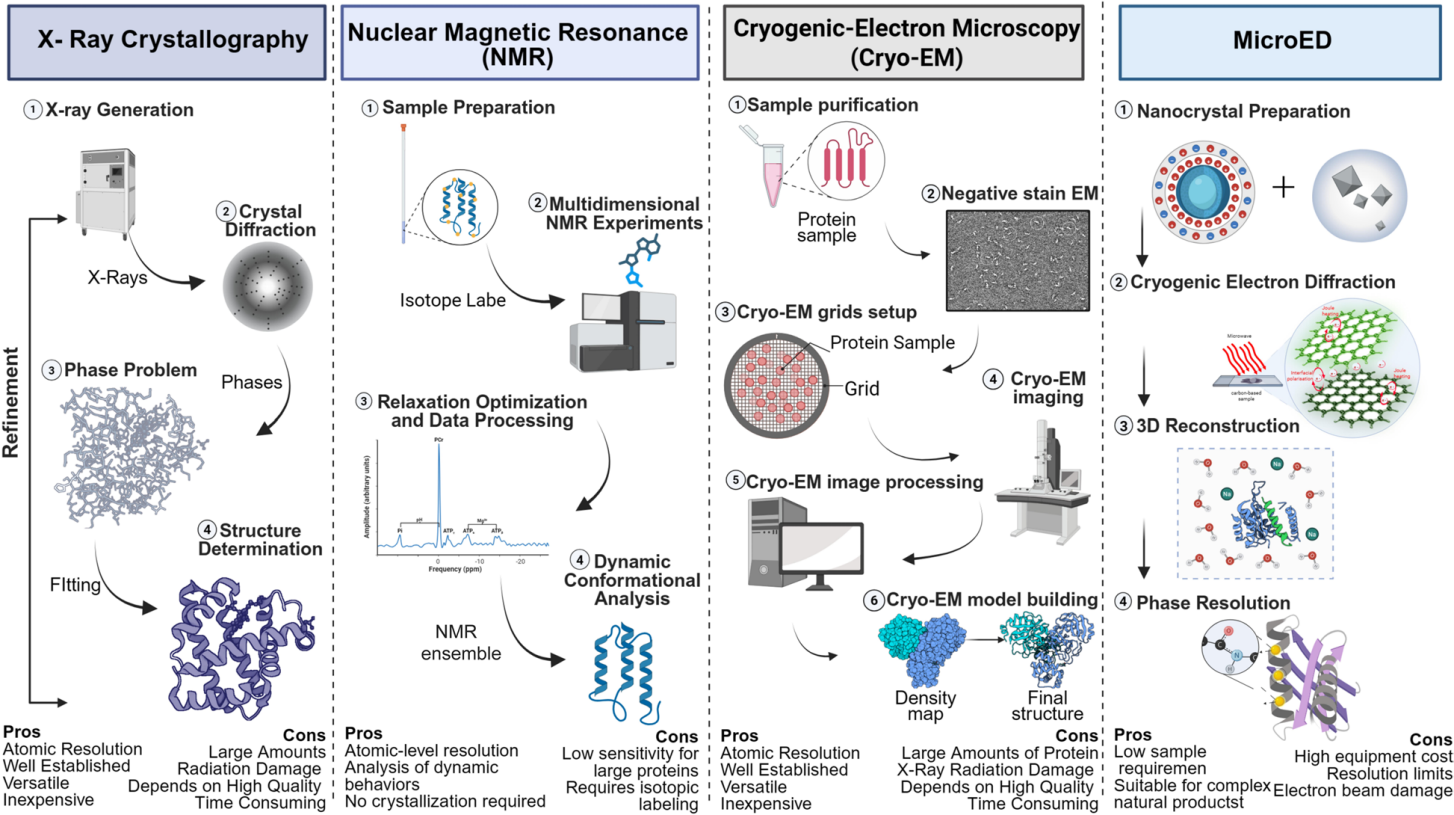
High-resolution structural biology tools. This figure provides an overview of key high-resolution structural biology tools for TCM structural pharmacology, focusing on X-ray crystallography, NMR, Cryo-EM, and MicroED. It highlights each tool’s role in solving TCM research challenges, such as analyzing flexible targets, capturing dynamic binding). processes. Together, these complementary techniques form an integrated pipeline for elucidating the mechanisms of active ingredients in TCM

The rapid development of high-resolution tools for structural biology has provided powerful support for addressing key challenges in the structural pharmacology of Chinese medicines. These include analyzing flexible target conformations, capturing dynamic binding processes, handling complex mixtures, and predicting interactions. Techniques such as X-ray crystallography, NMR, Cryo-EM, and microcrystalline electron diffraction (MED) complement each other to build a complete research chain from the atomic scale static structure to the dynamic behavioral characterization of biomolecules. This integrated approach provides a strong basis for uncovering the mechanisms of action of TCM active ingredients (Fig. 4).

#### Crystallography, X-Ray

In TCM research, X-ray has been successfully applied to analyze the interaction mechanism of various active ingredients with receptors, ion channels, and metabolic enzymes. A typical example is the structural analysis of shikonin, the core antiviral component of the TCM Ziziphus, in complex with the SARS-CoV-2 main protease at 1.8 Å resolution. The structural analysis clearly shows that the naphthoquinone moiety of shikonin precisely anchors the protease catalytic dyad His41-Cys145 through T-type π-π stacking, while its propionyl side chain occupies the S1, S2 and S4 sub-pockets, forming a unique sandwich binding configuration across the active center (Li et al. 2021). This discovery not only explains the atomic-level mechanism behind shikonin’s broad-spectrum anti-coronavirus activity but also provides a new idea for the design of variant inhibitors based on the natural naphthoquinone backbone. However, the main limitation of X-ray is its tendency to capture only the most stable, lowest-energy binding state, with limited ability to characterize transient intermediates or sub-stable states during the binding process. Time-resolved crystallography techniques combined with fast freezing can partially compensate for this shortcoming by capturing reaction intermediates.

#### Nuclear magnetic resonance spectroscopy, NMR

NMR spectroscopy has become an indispensable core technology for structural pharmacology studies of TCMs owing to its atomic-level resolving power in solution and its unique advantages for analyzing molecular dynamics. Methodological innovations have significantly improved the throughput and sensitivity for analyzing trace components in complex TCM systems, such as the recently developed relaxation-optimized non-uniform sampling strategy (Lin et al. 2023), combined with chromium-based relaxation reagent to significantly shorten the longitudinal relaxation time, combined with the compressed perception reconstruction technique, has reduced the two-dimensional quantitative NMR detection cycle for highly toxic diterpene alkaloids in the epiphyllums was compressed from the traditional 9 h to 30 min. This technique, for the first time, enables high-throughput, accurate quantification of microtoxicity in 18 batches of raw epiphyllums and their processed counterparts, thereby providing a molecular basis for the theory of the reduction of the toxicity of traditional herbal processing Table 1.

**Table 1:** Representative examples of structure-pharmacology relationships in TCM bioactive molecules

Class	Example Compound	Key Structural Features	Primary Target(s)	Pharmacological Action
Phenylpropanoid	Salvianolic Acid B	Catechol groups, carboxylic acid, planar arrangement	GJB2 (Connexin)	Inhibits HCC progression, modulates tumor microenvironment
Sesquiterpene Lactone	Eupalinolide B	α,β-unsaturated carbonyl, hydrophobic terpene ring	BCAT1	Inhibits BCAA metabolism, anti-cancer
Flavonoid	Baicalein	o-Diphenol hydroxyl groups, rigid planar structure	Hsp90 ATPase domain	Anti-inflammatory, antipyretic
Alkaloid	Berberine	Quaternary ammonium, conjugated rigid plane	AMPK, NF-κB p65	Anti-diabetic, anti-tumor
Metal Complex	Ru(III)-Schiff Base	Octahedral geometry, planar ligand moiety	DNA, Caspase-3	Pro-apoptotic, anti-tumor

This table provides a concise overview of the 'structure-determines-function' principle as discussed in the review

In the analysis of internal mechanisms, NMR can directly observe the interaction between TCM-derived small molecules and functional domains of nuclear receptors (MacTavish et al. 2025). Perturbation analysis of peramidoproton chemical shifts revealed the ligand-induced conformational-aggregation drift phenomenon and the molecular switching mechanism underlying transcriptional regulation. In the field of metabolic regulation, a recent study has successfully tracked the biosynthetic flux of phenolic acids in Salvia miltiorrhiza under methyl jasmonate stimulation using hydrogen-1 NMR spectroscopy coupled with multidimensional correlation spectroscopy (Li et al. 2024).

Despite these strengths, current NMR technology still faces limitations in resolving signals from large molecular weight proteins and capturing transient weak interactions. These challenges necessitate complementary approaches, such as in situ Cryo-EM structural analysis and molecular dynamics simulation, to construct all-atom models and fully elucidate complex biological mechanisms.

#### Microcrystal electron diffraction, Cryo-EM

The technological breakthrough of Cryo-EM lies in the direct capture of biological macromolecules in solutions without crystallization, with the advantages of a wide range of sample applicability, a large resolving scale, and a resolution up to the near-atomic level. The workflow relies on 200–300 kV high-resolution transmission electron microscopy (TEM) to capture single-particle projections, and maximum-likelihood 3D reconstruction algorithms such as RELION and CryoSPARC. By generating density maps with a precision of 2–3 Å, this workflow enables the construction of atomic models without symmetry constraints. Cryo-EM is especially suitable for analyzing membrane protein complexes, viruses, supramolecular machines, and other systems often challenging for conventional X-ray crystallography. In TCM target research, Cryo-EM has successfully analyzed the structure of the ternary complex formed by human ATP-citrate lyase and its inhibitor (Lyu et al. 2024). This study, for the first time, reveals the unique double-arrowed tetrameric conformation of ACLY and its dynamic assembly process. It also captured the inhibitor binding-induced conformational change of the key residues of Ile344-Arg379 and elucidates the mechanism of deformation inhibition through the "gating switch" mechanism, which is usually difficult to be observed in the static crystal structures. Furthermore, Cryo-EM has advanced mechanism research in the structural pharmacology of Chinese medicine by enabling high-resolution analysis of active ingredient in complex with target proteins. Taking puerarin as an example, Cryo-EM pinpoints its metastable regulatory pocket bound to the α1/γ2 subunit interface of the GABAA receptor reveals. The structure revealed its structural basis by stabilizing the receptor's desensitized state conformation through π-π stacking and hydrogen bonding networks, clarifying its role as an orthosteric metastable modulator for the enhancement of the GABAA current at the atomic level (Lyu et al. 2024).

Despite these advantages, Cryo-EM still faces sample preparation, especially for hydrophobic or low-molecular-weight proteins, which require high purity. In recent years, technology has developed rapidly: AI-driven algorithms have significantly improved data processing efficiency and dynamic modeling capabilities; sample processing technologies such as microfluidics have increased the data acquisition rate of membrane proteins; and in situ Cryo-EM has enabled the analysis within natural intracellular structures. These advances are pushing structural pharmacology from a single static structure analysis to a "dynamic functional conformation" research paradigm.

#### Microcrystal electron diffraction, MicroED

MicroED uses a high-energy electron beam as a probe to continuously collect diffraction patterns from rotating nanocrystals (< 200 nm). These patterns are analyzed by standard crystallography software to obtain atomic-level 3D structures. This technique combines the advantages of Cryo-EM and X-ray crystallography. MicroED requires only nanogram quantities of sample, and can analyze crystals as small as 50–200 nm, which is significantly below the > 10 μm threshold typically required for conventional X-ray crystallography. Consequently, MicroED is particularly suited for rapid structure elucidation of natural products that are difficult to crystallize, available in limited purity, or scarce in small quantities, thus dramatically shortening the discovery cycle of active precursors. For example, Rodríguez et al. obtained and validated the complete 3D structure of aspercyclinin A directly from the crude extracts of Aspergillus spp. without purification using MicroED. The analysis also confirmed the conformations of the known metabolites geodin and sulochrin (Xue et al. 2025), and unveiled a novel polycyclic polyketide scaffold embedded by distinct biosynthetic pathways. This finding provides a foundation for the development of anticancer drugs that exhibit both chemotherapeutic and immune-activating functions.

MicroED has demonstrated unique advantages in drug discovery, especially in structure resolution of difficult-to-crystallize targets, membrane proteins, and small molecules. With the advancement of automated data acquisition and phasing strategies, MicroED is expected to become one of the core methods for structure-directed drug design, accelerating natural product development, ligand optimization, and research on membrane protein drug targets.

### Computational approaches

**Fig. 5:**
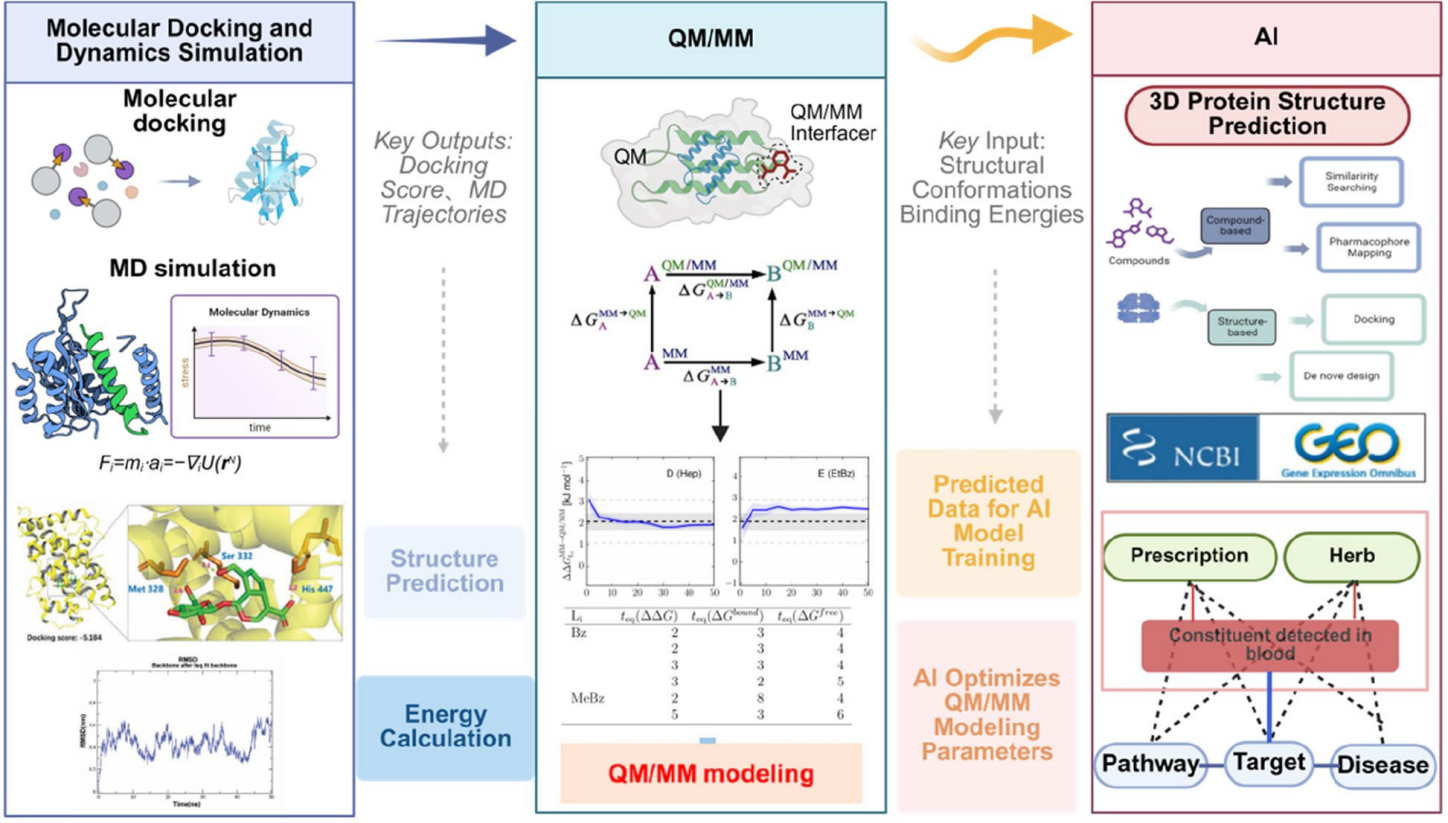
Calculation methods and process applications. This figure outlines core computational methods used in TCM structural pharmacology, including molecular docking, MD simulation, QM/MM, and AI, along with their integrated workflow. It shows key inputs (e.g., TCM prescription structures) and outputs (docking scores, binding energies, predicted structures). These methods simulate drug-target interactions, optimize energy calculations, and enable AI-driven target prediction/and compound screening, thereby supporting an iterative "experimental-computational" loops to decode TCM's multi-component-multi-target synergistic mechanisms

The core goal of structural pharmacology in Chinese medicine is to elucidate the synergistic mechanisms of multi-component, multi-target interactions (Fig. 5). Achieving this relies on a closed-loop research chain: experimental data drives computational modeling, and computational predictions guide experimental validation. Experimental techniques (as discussed in Section 3.1) offer crucial static and dynamic structural data, while computational methods enable comprehensive mechanism modeling through simulation, prediction, and screening, thus guiding experimental design. These two approaches work synergistically to advance research.

#### Molecular docking and molecular dynamics simulation, docking/MD

Molecular docking and kinetic simulation are the core technologies to analyze the multi-target synergistic mechanism of TCM. Molecular docking is based on the lock-and-key model and the induced fit theory. It predicts and evaluates the optimal binding conformation and affinity of a receptor’s active pocket, leveraging geometrical, energetic, and chemical environment complementarity. Kinetic simulation, on the other hand, simulates the dynamic behavior of molecular systems through Newtonian mechanical equations, revealing conformational changes and binding stability. The combination of these two approaches accurately predicts the targeting of herbal components and elucidates the dynamic regulatory processes. In the study of TCM, molecular docking successfully predicted that geniposidic acid (GPA) forms a "T-hammer" binding conformation with the farnesoid X receptor (FXR), similar to that of the known agonist. Notably, key hydrogen-bonding residues (Ser332, His447) were subsequently validated by targeted mutagenesis and biophysical experiments (Fan et al. 2025). In addition, based on the docking-predicted binding mode of Ponicidin and the Keap1 protein, further 50 ns MD simulations were performed. These simulations enabled an in-depth analysis of the dynamic stability of the Keap1-PGAM5 complex and revealed the molecular mechanism by which Ponicidin stabilizes the complex and regulates the mitochondrial apoptotic pathway. Despite the significant advantages of technology, it still faces bottlenecks such as limited computational accuracy and difficulties in modeling multiple targets. interaction. Future efforts should focus on integrating multi-scale modeling and AI technology, along with experimental validation, to enhance predictive reliability and biological relevance (Chen et al. 2024).

#### Quantum mechanics/molecular mechanics, QM/MM

The QM/MM method enables accurate simulation of electron transfer-involved processes, such as enzyme-catalyzed reactions. It achieves this by dividing the system into a high-precision quantum–mechanical (QM) region and an efficient molecular-mechanical (MM) region. This technique is particularly useful for revealing mechanisms of metabolic transformation of active ingredients and their subtle electronic interactions with targets in TCM. For example, in TCM metabolism studies, QM/MM clarified the oxidation pathway of dihydroxycoumarin metabolized by the human CYP3A4 enzyme. Calculations indicated that the method produces γ-ketoallyl aldehyde with an activation energy barrier of ~ 9.0 kcal/mol, compared to the higher barrier of ~ 25.0 kcal/mol for the epoxide pathway. This explains two experimental observations: only the γ-ketoallyl aldehyde is detected as a product, and the epoxide product is not observed. Additionally, this mechanism explains how γ-ketoallyl aldehyde inactivates the CYP3A4 enzyme. In target interaction studies, QM/MM has provided insight into weak interactions within the binding pocket of the candidate natural product VEGFR-2 inhibitor. These include anion-π interaction with Asp1046, π-π stacking with Phe918, hydrogen bonding between the ligand ureido group and Glu885, and front-line orbital complementarity (Yan and Hirao 2025). These atomic-level insights are crucial for designing high-affinity inhibitors based on electronic structures. However, the main challenges of QM/MM include high computational cost and sensitivity to parameter choices—such as QM region size and QM method selection. To alleviate these issues, recent advances include adaptive QM/MM partitioning algorithms that reduce errors introduced by manual partitioning, as well as AI model integration to improve efficiency and accuracy in quantum computations.

#### Artificial intelligence (AI)

Artificial Intelligence (AI) accelerates TCM structural pharmacology from experience-driven to data-driven research through multidimensional technology integration (Fig. 5). Chen Yuchian's team at Peking University developed the TCMBank database, which contains information on 9,192 herbs, 61,000 ingredients, and 15,000 targets. By using AI, knowledge mapping, natural language processing, and deep learning, researchers mine this data to build a multi-dimensional network linking TCM ingredients, targets, pathways, and diseases. To predict compound synergy mechanisms, the integrated computational protocol combining "molecular docking + hybrid neural network (HNN) drug-target affinity prediction + 50 ns molecular dynamics" was used for the formula Wen-Gao-Tang. This study revealed that soy cerebrosides interact with the low-density lipoprotein receptor (LDLR) via dual hydrogen bonding and hydrophobic interactions, thereby blocking LDL endocytosis. This effect further synergizes with curcumin to inhibit the nuclear translocation of hypoxia-inducible factor-1α (HIF-1α). The combination of soy cerebrosides and LDLR can block LDL endocytosis, and curcumin can inhibit HIF-1α nuclear translocation. In vitro experiments verified that this combination reduced macrophage inflammatory factor IL-6 by 42%, thereby quantifying, for the first time, the synergistic mechanism of the "lipid-protein-inflammation" axis of Wen-Gu-Tang. In another case involving Ganoderma Lucidum Spore Powder, the TCMBank-API was used to retrieve the 3D structure of triterpenoids. The company combined migration learning with a LoRA fine-tuned macro-language model (TCMLLM-PR) on 68,654 prescription data and predicted 7 triterpenoid side-chain modification sites that can improve intestinal permeability. Subsequent adjustment of the wet ball milling-liposome encapsulation process was adjusted accordingly, and the AUC₂80% in vivo of the rat was reduced by 1.4%. After the wet ball milling-liposome encapsulation process was adjusted accordingly, the in vivo AUC0-24 h in rats increased by 2.3-fold, and the production cycle was shortened by 30%, demonstrating the advantages of AI in process amplification. In addition, ChatDD multimodal modeling, AlphaFold for protein prediction, and molecular dynamics simulation developed by Tsinghua University accelerated the intelligent process from ingredient screening to preclinical studies in TCM research.

Despite these advances, AI applications in TCM face data quality and model interpretation challenges. Issues like inconsistent terminology and database annotations require standardized, collaborative approaches to improve data reliability. Most AI models struggle with dynamic interaction simulation, underscoring the need to incorporate structural biology experimental data for comprehensive validation. Collaborative platforms such as the AI-Systems Biology Joint Platform, which integrate multi-omics, microbiota, and clinical data, offer interpretable models that enable cross-scale validation. These efforts illustrate the intersection of computational methods, biology, and clinical research in TCM.

To modernize TCM with AI, priority should be given to forming a seamless 'data-algorithm-experiment' loop by strengthening standardized data systems, developing interpretable algorithms, and confirming predictions through experimental validation. AI discoveries will be advanced to clinical validation, driving TCM drug innovation, facilitating internationalization, and establishing a paradigm built on intelligent prediction, multi-technology validation, and industrial application.

## Technical bottlenecks in structural pharmacology of TCM

The modernization of TCM's structural pharmacology is profoundly transforming the scientific paradigm of TCM research from "empirical induction" to "dynamic design". The core is to reveal the dynamic interactions among multiple components and targets in TCM through the integration of multidimensional technologies, and to promote the scientific interpretation of the synergistic effect of compounding. Although the deep integration of AI, multi-omics integration, and dynamic simulation technology has injected new momentum into the discipline's development, the current research still faces many systematic challenges.

### Changes in protein dynamics are difficult to confirm

Conformational drift of disease-related proteins directly determines their gain of function or inactivation, thereby drives pathological processes. However, current technologies remain insufficient to completely capture this dynamic chain, which has become a bottleneck in structural pharmacology of Chinese medicine.

#### Protein conformational dynamics and the absence of causal chains in disease

The study of protein conformational dynamics is essential for understanding molecular mechanisms of disease. For example, the conformational transition of amyloid from soluble α-helical monomers to β-folded oligomers is a molecular switch for early synaptic toxicity in Alzheimer's disease (Aβ42) and Parkinson's disease (α-synuclein). These diffusible oligomers disrupt cellular membrane integrity, induce intracellular Ca^2^⁺ imbalance, and trigger ROS bursts, ultimately leading to apoptosis (Gonzalez-Garcia et al. 2021; Sun et al. 2021). In KRAS-G12D mutant pancreatic cancer, the spontaneous flip frequency of the Switch-II region from "active" to "inactive" conformation decreased by 4.7-fold, resulting in sustained activation of the RAF-MEK-ERK pathway and enhanced tumor migration (Li et al., 2025). Similarly, the transition of STAT3 from the "inhibitory" to the "active" conformation is closely related to the over-amplification of the IL-23/IL-17 axis (Zhu et al. 2024). in keratinocytes of psoriatic lesions (Zhu et al. 2024). However, due to the extremely short lifetimes of millisecond intermediate states and the inability to capture conventional static structures (X-ray crystallography, Cryo-EM static snapshots), it has been difficult to establish a complete "conformation-disease" causal chain as described above.

#### Modulation of drug efficacy by protein conformational dynamics

The efficacy of active ingredients in TCM depends on their interactions with target proteins, and protein conformational dynamics significantly modulate binding modes and the regulatory efficacy of drugs. For example, tretinoin was shown to target the "non-DNA-binding" conformation of p65, block NF-κB nuclear translocation, and reverse the resistance of triple-negative breast cancer to adriamycin, resulting in a 2.8-fold decrease in IC₅₀ (Szczepny et al. 2018). In another case, baicalein increased the percentage of "inactivated" conformations of EGFR-T790M mutants from 38 to 71%, restoring gefitinib sensitivity and reducing tumor cell survival by 52% (Chen et al. 2022). Furthermore, AI-driven drug repositioning based on pathway profiling revealed that Tanshinone IIA inhibits oxidative stress-induced hepatic fibrosis by stabilizing the "closed" conformation of Keap1 and enhancing its ubiquitinated degradation of Nrf2, a prediction validated by HDX-MS.

#### Limitations of existing technology

Current techniques used to resolve dynamic changes in proteins (NMR, Time-Resolved Wide-Angle X-Ray Scattering (TR-WAXS), X-ray Crystallography, etc.) can investigate the flexibility of different regions of proteins, high-resolution three-dimensional structures, the equilibrium of conformational isoforms, and conformational changes and dynamic behavior during ligand binding. However, each of these techniques has significant limitations: liquid/solid NMR is limited by molecular weight size or time window; TR-WAXS lacks atomic-level structural details. Additionally, XFEL-TR-SFX is rare and low-throughput, and single-molecule fluorescence resonance energy transfer (smFRET) requires labeling and provides mainly one-dimensional distance information, and the chemical cross-linking step of XL-MS may interfere with the natural conformation of proteins. To overcome the "time–space-sensitivity" bottleneck and fully characterize the dynamic interactions between TCM components and proteins, it is necessary to integrate diverse technological tools. Combining techniques such as NMR, XFEL, Artificial Intelligence Molecular Dynamics Simulation (AI-MD), and physiologically relevant models, such as organoid or single-cell models, will provide a more comprehensive and biologically meaningful understanding of protein conformational dynamics in complex herbal formulations.

### Limitations of dynamic structural parsing

Although the dynamic combination of TCM components and flexible targets is the core mechanism underlying multi-target synergy, they remain challenging to fully capture due to the triple characteristics of "transient-weak interaction-multi-configuration". Existing technologies are mainly limited by three bottlenecks, namely, temporal resolution, conformational coverage, and multi-component modeling.

#### Kinetic blindness of transient binding

The binding of active ingredients in TCM to their targets often occurs within milliseconds or less and is dominated by weak interactions, such as hydrophobicity and hydrogen bonding (Kd in the micromolar range), resulting in transient binding events and weak signals. For example, the activity of flavonoids is indirectly regulated through the AMPK signalling pathway. Conventional techniques such as surface plasmon resonance (SPR) or isothermal titration calorimetry (ITC) lack the temporal resolution to capture conformational transitions on the millisecond-to-microsecond scale (Yu 2008). Conventional experimental techniques are therefore difficult to capture and detect stably, let alone measure, such fast and weak transient binding events due to their limited temporal resolution.

#### Resolving gaps in multiple conformational states of flexible targets

Flexible target proteins such as GPCRs and kinases often exist in multiple conformational states, and TCM components often produce "synergistic-antagonistic" effects across multiple targets. For example, tretinoin can simultaneously inhibit NF-κB and activate the Nrf2 pathway. Therefore, constructing a dynamic regulatory network to fully capture such multi-target mechanisms requires integrating multidimensional data, including genomic and proteomic profiles. However, conventional techniques such as X-ray crystallography and NMR have limited resolution in resolving the dynamic conformational changes of flexible targets. As a result, it is difficult to fully reflect the dynamic conformational changes in interactions between TCM components and flexible targets solely through conventional detection.

#### Difficulty in predicting the structural basis of multicomponent systems in Chinese medicine

The multi-component, multi-target, synergistic nature of TCM compounding as a core form of practice makes it particularly challenging for elucidating the precise mechanisms and structural basis underlying its interactions with dynamic biological targets.

##### Data scarcity and model bias

TCMs exhibit complex compositions, numerous targets of action, and interactions characterized by dynamic, spatiotemporally variable properties. These features make it difficult to build a comprehensive, highly accurate prediction model. Existing bioinformatics and computational methods, such as network pharmacology, require substantial experimental data for training and validation. However, there is a relative paucity of current studies on the in-depth mechanisms of TCM-dynamic target interactions, and the available data are scarce and poorly standardized. These limitations affect the predictive ability of the models, leading to inaccurate prediction of the dynamic target structure and a high susceptibility to overfitting, where models perform well on the training data but generalize poorly in real applications, which in turn hampers an in-depth understanding of the overall mechanism of TCM (He et al. 2024).

##### Complexity of modes of action

The synergistic effects of TCMs, which involve multiple components, targets, and pathways, reach beyond the traditional "single target" paradigm. Even advanced tools like network pharmacology struggle to fully characterize their complex spatial–temporal interactions (Zhao et al. 2023). Specifically, the physical processes by which herbal components bind—either synergistically or competitively—to dynamic targets and subsequently influence their conformation and function remain poorly understood (Fig. 5).

In summary, analyzing the dynamic binding mechanisms between TCM components (especially in compound systems) and flexible targets: the detection bottleneck of transient binding, the difficulty of capturing the dynamic conformation of targets, and the dilemma of structural modeling of multi-component systems. To overcome these limitations, it is necessary to integrate a variety of cutting-edge technologies, such as high-temporal-resolution imaging, Cryo-EM, AI-driven molecular dynamics simulation, and advanced mass spectrometry, and to combine them with physiologically relevant models, such as organoid/single-cell systems. This will facilitate the establishment of a cross-scale, multimodal research system, thereby breaking the "time–space sensitivity" bottleneck and enabling comprehensive analysis of the dynamic binding mechanism.

## Analyzing protein dynamics and future directions in complex systems of traditional Chinese medicine

**Fig. 6:**
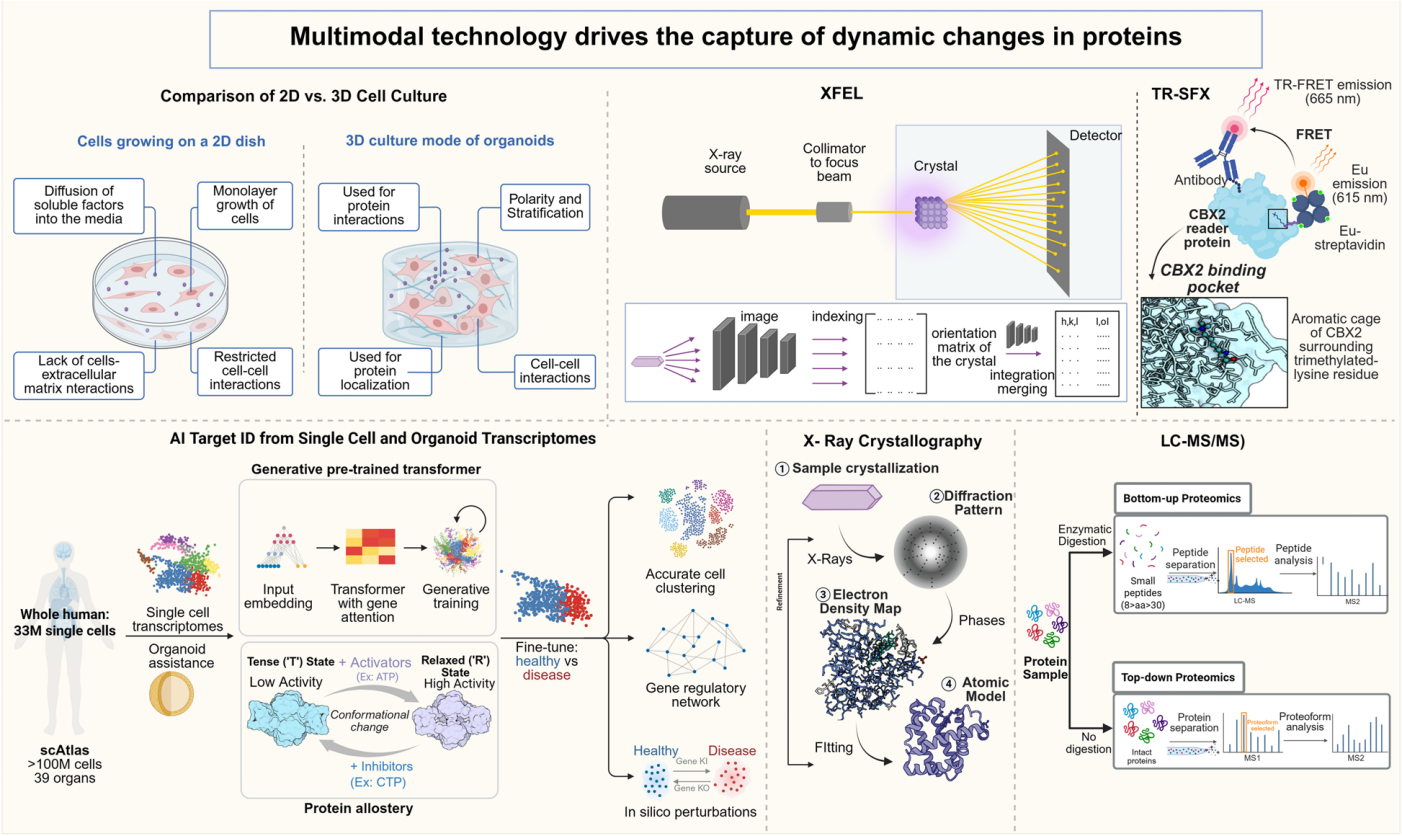
Multimodal techniques driving the capture of dynamic changes in protein structures. This figure illustrates a multimodal technique for analyzing TCM-related dynamic protein structures, including organoid and single-cell technology, time-resolved structural biology techniques (e.g., XFEL, TR-SFX), and AI modeling. It illustrates how these techniques synergize: organoids provide physiologically relevant models, time-resolved tools capture real-time conformational changes, and AI predicts dynamic structures supporting the decoding of TCM-protein dynamic interaction networks

Proteins are not static structures, and their biological activity depends on dynamic structures that exhibit varying degrees of heterogeneity in solution or in biofilms (Grandori 2023). Thus, the challenge for structural biology is to capture the structural dynamics of proteins under equilibrium or dynamic conditions. This involves transitioning from a single static image to a dynamic movie of a collection of conformations (Xie 2020). Ideally, these observations should be carried out in vitro and in vivo, within the natural environment. Time-resolved structural biology techniques allow researchers to observe structural changes in biomolecules on different time scales to understand their dynamic functions (McCafferty et al. 2020). The development of Cryo-EM technology has enabled the study of protein assemblies in a near-native state (Sun and Nissen 2024). Time-resolved X-ray crystallography and free-electron laser (FEL) technology can capture structural changes on the nanosecond to millisecond time scale. In situ and in vivo structural biology studies, such as single-particle tracking and high-resolution fluorescence microscopy, can provide structural data in a cellular environment or in vivo, minimizing artifacts introduced by extraction and purification and providing more physiologically relevant information (Gayathiri et al. 2023). The integration of multiple techniques offers a more comprehensive understanding of biological dynamics. To resolve the structural information of the multi-components of TCM, more advanced spectroscopic and mass spectrometric techniques, such as MS/MS and LC–MS, can be utilized in combination with NMR techniques (Zhao et al. 2023) (Fig. 6). These techniques can provide more comprehensive and precise structural information, which can help to understand the complex components of TCM and their interactions. In addition, the development of computational methods, such as molecular dynamics simulations and docking, serves as a powerful tools for investigating protein–ligand interactions and interpreting experimental data within a dynamic framework (Viscido et al. 2021).

### Organoid-single-cell technology coupled to analyze the dynamic evolution of proteins

Organoids, derived from stem cell self-organization, preserve cellular heterogeneity, genetic background, and organ function in vivo in a three-dimensional microenvironment, providing a physiologically relevant in vitro model for drug mechanism and toxicity studies. Single-cell RNA sequencing (scRNA-seq) and single-cell proteomics enable real-time tracking of gene and protein expression at the cell-subtype level. Together, these technologies allow multidimensional analysis of protein conformational changes across spatial, temporal, and functional domains during disease processes. For example, liver tissue can express CYP450 enzymes, and single-cell RNA sequencing has revealed that those with high CYP3A4 expression are more susceptible to artemisinin derivatives, providing a cellular basis for metabolic subtype identification in TCM research. In pancreatic ductal adenocarcinoma-like organoids, scRNA-seq identified a subpopulation of CD44⁺/ALDH1⁺ cancer stem cells, which showed a higher survival rate of 37% ± 5.2% under gemcitabine treatment, suggesting that drug resistance is associated with activation of the Wnt/β-catenin pathway. Single-cell karyotyping in colorectal cancer carcinoids confirmed the presence of distinct subclones immediately prior to treatment rather than drug induction, enabling prediction of radiotherapy resistance and guiding personalized herbal combination strategies. In neurodegenerative brain organoids, combining Fluorescence Lifetime Imaging Microscopy (FLIM) with scRNA-seq enables real-time monitoring of Aβ42-induced mitochondrial stress and cell-cycle block, providing a temporal window for screening neuroprotective herbal agents.

Single-cell technologies can analyze gene expression profiles and cellular heterogeneity. ScRNA-seq can reveal the evolutionary state of cells and differences between populations, identify unique cell subtypes and states, and reveal regulatory relationships, targets, and molecular mechanisms in disease processes. In a breast cancer single-cell mapping study, scRNA-seq identified 12 epithelial cell subtypes, among which an ERBB2 + subpopulation with constitutive activation of the EGFR/PI3K pathway exhibited a 3.2-fold reduction in paclitaxel sensitivity (Ali et al. 2024).

The combination of organoid and single-cell technologies allows researchers to analyze protein dynamics during disease development with unprecedented precision. By performing single-cell analyses in complex organoid environments, we can more accurately understand the role of protein dynamics in disease. Recent advances in FLIM technology enable real-time monitoring of organoid metabolic state, enhancing functional insights into protein dynamics and drug responses.

### Time-resolved structural biology resolves complex dynamics

Time-resolved structural biology addresses the limitations of traditional Cryo-EM's static snapshots. By employing X-ray free-electron lasers (XFELs) and serial femtosecond crystallography (SFX), researchers can capture protein conformational changes over femtosecond to minute timescales (Fig. 6).

The introduction of XFELs has revolutionized structural biology by producing extremely short (femtosecond) and intense X-ray pulses. This allows diffraction data to be collected from biomolecules before radiation damage occurs (Müller et al. 2024), embodying a "destroy first, image later" approach that permits study of biomolecules at room temperature and brings experiments closer to physiological conditions (Müller et al. 2024). TR-SFX, a major application of XFEL, involves triggering a reaction with a laser or hybrid device and subsequently probing it with XFEL pulses. This method enables observation of protein dynamics at timescales ranging from femtoseconds to minutes (Park et al. 2024). While first applied to photoactive proteins, TR-SFX can potentially investigate a broader spectrum of biomolecular systems (Caramello et al. 2024). For instance, German researchers used TR-SFX to capture seven temporal structures of the bacterial photosensory pigment Agp2-PAiRFP2, spanning from the PFR to the Meta-F state, revealing connections between chromophore isomerization, proton transfer, and protein conformational shifts (Grieco et al. 2024).

### AI modeling database-assisted protein dynamic structure prediction

AI is playing an increasingly important role in biological big data parsing (Zcelik et al. 2024; Guo et al. 2023). In structural biology, AI and machine learning (ML) techniques are rapidly advancing the study of intrinsically disordered proteins (IDPs) (Ramanathan et al. 2021). Through deep learning and other techniques, it is possible to establish dynamic structure prediction models of proteins or of complex system components in TCM (Aziz et al. 2024). Modeling protein dynamics and AI algorithms can help simulate the biological system dynamics and greatly improve the understanding of protein conformational relationships (Hou et al. 2024).

Furthermore, AI facilitates the integration of multi-omics data to identify biomarkers and therapeutic targets, thereby accelerating the drug development process (Li et al. 2015). AI-driven biomedical genomics research is widely used to process and analyze high-dimensional genomic data, promoting biomarker discovery and enhancing genome sequence annotation by integrating biomedical knowledge into algorithm development. AI-based biomedical genomics research can be divided into the following steps: data collection and preprocessing, algorithm development, verification and validation, and integration of biomedical knowledge (Aziz et al. 2024).

Combining TCM research with modern bioinformatics enables a deeper mechanistic understanding of herbal medicine through database screening and biological network analysis (Kong et al. 2024). For example, by constructing the component-target network of TCM, the multi-component and multi-target action characteristics of TCM can be revealed, providing valuable insights for novel drug development (Kong et al. 2024).

In conclusion, the future direction of analyzing the dynamic structure of proteins and the complex system of TCMs requires the integrated use of a variety of technologies and methods, including time-resolved structural biology, in situ/in vivo structural biology, spectroscopy, and mass spectrometry technology coupling, integration of organoid/organ microarrays and single cell-multi-omics, and AI modeling. Through the continuous development and application of these technologies, it is expected that a deeper understanding of the dynamic structure of proteins and the complex mechanism of TCM will ultimately provide new ideas and methods for drug discovery and therapeutic innovation (Fig. 6).

## Conclusion

Structural Pharmacology of Chinese Medicine is an interdisciplinary field that focuses on the "three-dimensional structure of chemical components, dynamic conformation of biological macromolecules—pharmacological network-clinical phenotype" as its core and systematically interprets the "multi-component-multi-target-multi-pathway" mechanism underlying TCM efficacy. Its mission is to translate the traditional "holistic view" into verifiable and designable molecular language using modern structural biology and computational chemistry methods.

The field has constructed a closed-loop framework of "quantum chemical computation, molecular docking/dynamics, Cryo-EM/time-resolved crystallography, organoid-single-cell validation, and clinical cohort correlation", which enables cross-scale analysis from atoms to organisms and provides reproducible experimental paradigms for the mechanism of synergistic compounding.

However, the development of structural pharmacology of Chinese Medicine is still constrained by three major bottlenecks: firstly, the structure-target gap is prominent, only about 30% of the active ingredients in the existing database have resolvable 3D target information, resulting in many potentially efficacious molecules lacking mechanistic annotations. Secondly, the dynamic capture capability is insufficient; transient binding events involving millisecond-level conformational flips and micromolar-level weak interactions pose an extreme challenge for existing time-resolved experimental techniques. Finally, the lack of data standardization, batch differences in herbs, non-uniformity of experimental parameters, and fragmentation of heterogeneous data have seriously weakened the generalization ability and clinical transferability of AI models, hindering the transition from "empirical prescription" to "precise design".

To address these challenges, we propose an integrated strategy based on "multimodal fusion and data standardization". Firstly, we build a structural-dynamic closed-loop platform based on TCMBank 2.0, which systematically integrates real-time conformational data from HDX-MS, TR-SFX, and smFRET. The system introduces the dynamic prediction engine of AlphaFold-Multimer, which can increase the coverage of the three-dimensional targets of the active ingredients of TCM from about 30% to more than 60%. Secondly, the molecular mechanism of GPCR-arrestin bias activation is elucidated for the first time using the joint study of Xiexin Decoction and synthetic microbiomics as a standardized example, laying the foundation for templating compounding data. In addition, the 2D-LC/IM-MS metabolic flow platform was deployed to enable simultaneous quantification of more than 3,000 ingredients, facilitating the construction of a spatiotemporally resolved database of pharmacological substances. Finally, with the help of light sheet fluorescence microscopy (LSFM), the dynamic shuttling of paeoniflorin at the lysosome-mitochondria interface is tracked in real time with a temporal and spatial resolution of 50 nm/30 fps in the model of Gui Zhi Fu Ling Pill, which provides visual evidence for the theory of the "sovereign-minister-assistant-envoy", and supports cross-scale mechanistic standardization.

With the continuous iteration of AI-MD, synthetic biology, and multimodal dynamic analysis platforms, structural pharmacology of Chinese Medicine is expected to play a key role in interpreting the mechanism of "multi-component-multi-target-dynamic synergy". The new generation of precision drug discovery and the formulation of international standards. These developments will establish a predictable and scientific foundation for the modernization of TCM.

The modernization of TCM is challenging because it is difficult to analyze the molecular mechanism and understand the complex component system. The complexity of TCM compounding, the challenges in protein dynamic analysis, and the limitations of technological capabilities in mechanistic verification have collectively impeded the development of understanding the components and targets. Existing studies show that only about 30% of the active ingredients in TCM have a clear chemical structure and a corresponding biological target (Gong et al. 2024). Although the BATMAN-TCM 2.0 database enhances the data volume of component-target interaction of TCM to 1.5 times by integrating the literature mining and prediction models, problems such as insufficient 3D structural information and missing dynamic interactions data persist. Multimodal fusion technology provides promising solutions. The latest study used laser-induced breakdown spectroscopy combined with deep learning to realize rapid identification of the origin of Chinese herbal medicines, with an accuracy of 92.3% (Yang et al. 2022). This spectral-image bimodal approach provides a technical paradigm for the standardized characterization of TCM structure databases. Recent studies have shown that combining structural databases with cross-border research has achieved remarkable results in modernizing Chinese medicine.

At present, some cutting-edge dynamic structure capture techniques have been widely used in the analysis of TCM compound-dynamic targets, e.g., two-dimensional liquid chromatography-ion mobility mass spectrometry (2D-LC/IM-MS enables simultaneous dynamic monitoring of over 3,000 ingredients in a compound. For example, the study of Qingluo Drink for the treatment of rheumatoid arthritis showed that the metabolic half-life of its 16 active ingredients in liver microsomes varied by 4 orders of magnitude (Zheng et al. 2022). This technology breaks through the limitations of traditional "static fingerprinting" and establishes a spatially and temporally resolved basic database of medicinal substances. Moreover, deep learning-based LSFM visualizes the dynamic distribution of herbal ingredients at the cellular scale. In the study of the anti-tumor mechanism of Gui Zhi Poria Pill, the technology successfully captured the dynamic shuttle process of paeoniflorin at the lysosome-mitochondria interface with a resolution of 50 nm, 30 fps (Xie et al. 2025). Dynamic structure capture technology is promoting research on Chinese medicine compounding from a two-dimensional "component-target" perspective to a four-dimensional "space–time-function" analysis. This paradigm change not only reveals the dynamic scientific connotation of the theory of "sovereign, minister, assistant, envoy", but also provides methodological support for the precise analysis of the complex mechanism of "multi-component-multi-target-dynamic synergy". With continuous optimization of the multimodal dynamic analysis platform, research into the modernization of TCM will enter a new stage of predictability and regulation.

The integration of AI, multimodal dynamic analysis technology, and synthetic biology represents a significant development in the field of structural pharmacology of Chinese medicines. This integration will undoubtedly lead to a series of breakthroughs, with the potential to elucidate the nature of the action of Chinese medicines as “multi-component-multi-target-dynamic synergism”. Furthermore, TCM is positioned for transformative growth, fully elucidating the nature of multi-component, multi-target, dynamic synergism and contributing uniquely to next-generation drug discovery and global health through TCM resources.

## Data Availability

The data supporting the findings of this review are available from public databases, including TCMBank and BATMAN-TCM 2.0. Additional relevant data can be retrieved from the cited references in this review. No unpublished original data were generated in this study.

## Abbreviations

AI: Artificial Intelligence
APL: Acute Promyelocytic Leukemia
Cryo-EM: Cryo-Electron Microscopy
EB: Eupalinolide B
FEL: Free Electron Laser
FLIM: Fluorescence Lifetime Imaging Microscopy
FXR: Farnesol X Receptor
GPA: Geniposidic Acid
GPCR: G Protein-Coupled Receptor
HDX-MS: Hydrogen-Deuterium Exchange Mass Spectrometry
IDP: Intrinsically Disordered Protein
ITC: Isothermal Titration Calibration
LSFM: Light Sheet Fluorescence Microscopy
MD: Molecular Dynamics
MED: Microcrystalline Electron Diffraction
MIRI: Myocardial Ischemic Reperfusion Injury
NMR: Nuclear Magnetic Resonance
PPA: Phenylpropanoid
QM/MM: Quantum Mechanics/Molecular Mechanics
SAB: Salvianolic acid B
scRNA-seq: Single-Cell RNA Sequencing
smFRET: Single-Molecule Fluorescence Resonance Energy Transfer
SPCM: Structural Pharmacology of Chinese Medicine
SPR: Surface Plasmon Resonance
TCM: Traditional Chinese Medicine
TEM: Transmission Electron Microscopy
TR-SFX: Time-Resolved Serial Femtosecond Crystallography
TR-WAXS: Time-Resolved Wide-Angle X-Ray Scattering
UGCG: UDP-glucose ceramide glucosyltransferase
XFEL: X-ray Free Electron Laser
